# X-ray CT Analysis of the Cross-Section of a 3D-Printed Deformed Layer

**DOI:** 10.3390/ma14247764

**Published:** 2021-12-15

**Authors:** Ho-Jae Lee, Eun-A Seo, Won-Woo Kim, Jun-Mo Yang, Jae-Heum Moon

**Affiliations:** 1 Department of Structural Engineering Research, Korea Institute of Civil Engineering and Building Technology Daehwa-Dong, Goyang-si 10223, Gyeonggi-do, Korea; sea0524@kict.re.kr (E.-A.S.); kimwonwoo@kict.re.kr (W.-W.K.); mjh4190@kict.re.kr (J.-H.M.); 2 Department of Civil Engineering, Keimyung University, 1095 Dalgubeol-daero, Dalseo-gu, Daegu 42601, Korea; jm.yang@kmu.ac.kr

**Keywords:** 3D concrete printing, layer cross-section, layer deformation, X-ray CT, compressive strength

## Abstract

In this study, we experimentally analyzed the deformation shape of stacked layers developed using three-dimensional (3D) printing technology. The nozzle traveling speed was changed to 80, 90, 100, and 110 mm/s when printing the layers to analyze its effect on layer deformation. Furthermore, the cross-sectional area and the number of layers were analyzed by printing five layers with overall dimensions of 1000 (w) × 2200 (l) × 50 (h) mm (each layer was 10 mm high) using Vernier calipers. Moreover, we analyzed the interface and cross-sectional area of layers that are difficult to confirm visually using X-ray computed tomography (X-ray CT) analysis. As a result of measuring the deformation at the center of the layer, it was confirmed that the deformation was greater for lower nozzle traveling speeds. Consequently, the X-ray CT analysis verified that the layer had the same cross-sectional area irrespective of the layer printing order at the same nozzle travel speed, even if the layer was deformed.

## 1. Introduction

In recent years, additive manufacturing (AM) technology has become a practical alternative as a precise manufacturing technology in the rapid prototyping, aerospace, jewelry, and biomedicine industries, among others. Furthermore, AM is being adopted in fields involving the manufacturing of large objects, such as in construction and defense [1,2,3,4,5]. While AM techniques are applied in methods such as binder jetting, powder bed fusion, and material jetting, the material extrusion method is most widely used in the current three-dimensional (3D) printing market when considering it mainly employs thermoplastic (polymeric) materials, which can be easily deformed when the heating temperature exceeds the glass transition temperature [6,7,8,9].

Three-dimensional construction printers widely use digitally fabricated concrete, which is produced using a composite-based cementitious binder, as a core material because it exhibits high strength, low cost, and high fluidity after mixing and before setting, thereby allowing easy deformation through extrusion. The constituent material is further derived by mixing the cementitious binder, aggregate, water, and other ingredients. Once these materials are mixed sufficiently, they behave as Bingham fluids before they are initially set [10,11,12,13,14,15,16]. When concrete exhibiting Bingham fluid behavior is used as an AM material, it behaves like a Bingham fluid when a force greater than the yield stress is applied while it is being pumped and extruded, and it is capable of maintaining its shape after lamination, owing to its plastic viscosity [16,17].

However, concrete maintains its fluidity longer than the thermoplastic materials used in material extrusion, making it difficult to control the setting and hardening time of thermoplastic materials, owing to environmental factors (temperature, humidity, and direct sunlight), types of materials, and the composition ratio. Therefore, during continuous lamination, the previously laminated layer does not harden and results in deformation due to the load and extrusion pressure of the upper layer. This made predicting the exact dimensions of the layer difficult. In previous studies, the shape of the layer was verified by measuring its width and height after printing [18], followed by simulations [19,20]. Although a precise and accurate layer prediction is essential for designing an actual print, there is a lack of research on suitable techniques for accurate dimension prediction.

**Figure 1:**
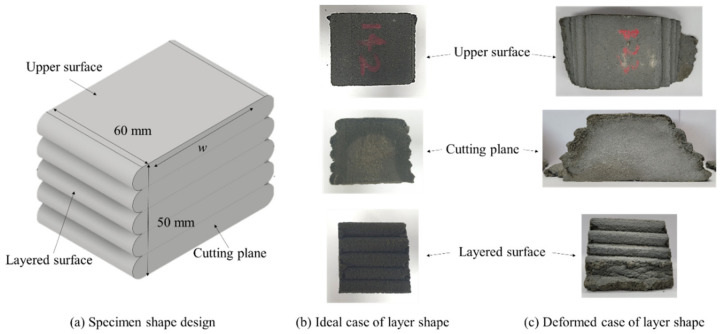
Specimen shape. (**a**) Overall design, (**b**) ideal case, and (**c**) deformed case.

When a specimen with a 3D-printed layer is designed as shown in Figure 1a, its shape can be developed as shown in Figure 1b if the layer is successfully printed. However, in reality, deformed specimens are often developed, as shown in Figure 1c. Lee et al. [18] conducted a study that confirmed the deformation phenomenon shown in Figure 1c, where they found that the extrusion rate of the material was the same as that during the printing process.

**Figure 2:**
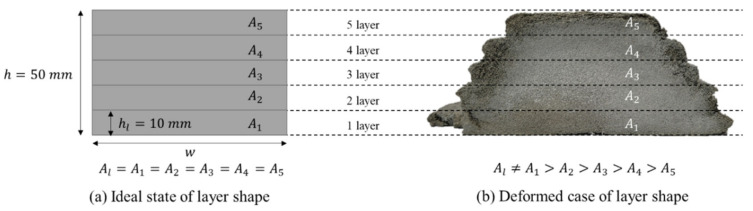
Cross-sectional area of the layer. (**a**) Ideal and (**b**) deformed cases.

If the nozzle traveling speed, layer height, and extrusion rate is the same during the printing process, the cross-sectional area of all layers should be the same, as shown in Figure 2a. However, in the actual shape, the width of the layer was wider when considering that another layer was located below it, as shown in Figure 2b. Herein, the total height of the printed layer was originally designed. Considering the cross-sectional area of the layer, although the lower layer should be wider, such a phenomenon rarely occurs because all conditions are the same during the printing process.

The purpose of this study was to analyze the cause of layer deformation in the 3D printing technique using cementitious materials and the tendency of interface change due to layer deformation. In addition, the method for analyzing the effect of the deformation of the layer on the cross-sectional area of the layer was aimed at comparing the results using the external investigation method and the X-ray CT (X-ray Computed Tomography) method.

The purpose of measuring the cross-sectional area of a layer can be explained with the example of Figure 2. As shown in Figure 2b, when the layer width is measured based on the edge protrusion of each layer, it can be seen that Layer 1 is the widest and Layer 5 is the narrowest. Furthermore, if the height of the layer is measured based on the inward portion between the upper and lower protrusions along the black dotted line in the figure, the height of the layer appears to be almost the same. As shown in Figure 2b, when the cross-sectional area of a layer is estimated using the width and height measured at the outside of the layer, it can be predicted that the cross-sectional area of Layer 1 is the largest and the cross-sectional area of Layer 5 is the narrowest.

However, according to previous studies, it is known that the main factor affecting the cross-sectional area of a layer is the amount of extrusion [21,22,23]. Therefore, when outputting with the same amount of extrusion, the layer should have the same cross-sectional area regardless of the layer’s shape. Although the amount of extrusion decreased with time in the previous study, the reduction in extrusion and the cross-sectional area of the layer occurred when printing more than seven layers. In this study, the moving speed of the nozzle was found to be a factor affecting the width of the layer [23].

Therefore, in this study, by setting the moving speed of the nozzle, not the extrusion amount, as a factor affecting the cross-sectional area of the layer, a study was conducted to fabricate the specimen and analyze the cross-section. In order to calculate the cross-sectional area, the method of measuring the apparent width and height was used as described in Figure 2, and X-ray CT was used for the analysis of the interface as used in previous studies [24,25,26]. After analyzing the interface using X-ray CT, we compared the appearance measurement method and the method of calculating the cross-sectional area using X-ray CT analysis as a method of calculating the cross-sectional area of the layer based on the interface.

## 2. Experimental Method

### 2.1. Printing Conditions

#### 2.1.1. Testing Equipment and Nozzle

**Figure 3:**
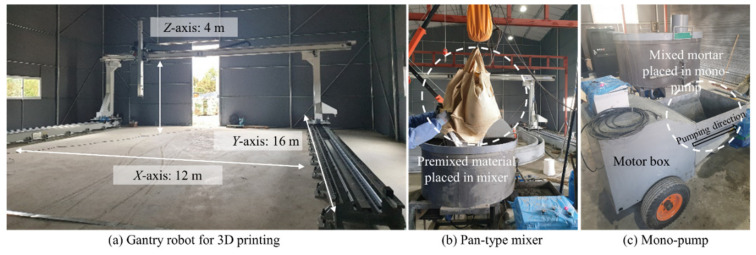
Printing design for the experimental test [24].

Figure 3 shows the equipment used in this study, manufactured by our team and used in a previous study [23]. The equipment comprised of a 7.5 kW batch-type mixer, a 7.5 kW mono-pump, a 20 m-long single-wire-reinforced high-pressure hose, and a circular nozzle with a 25 mm diameter. A 7.5 kW pan mixer capable of mixing over 150 × 10^3^ cm^3^ (150 L) per batch was used for the mortar mixer. The pump used a 7.5 kW motor and a rotor-stator with a maximum pressure and a grain size of 4 N/mm^3^ and 6 mm, respectively. The single-wire-reinforced high-pressure hose was connected to a mono-pump. In the test, a gantry robot (12 (*x*-axis) × 16 (*y*-axis) × 4 (*z*-axis) m) was used as the 3D positioning equipment. 

#### 2.1.2. Laminated Specimen with Nozzle Traveling Speed

**Figure 4:**
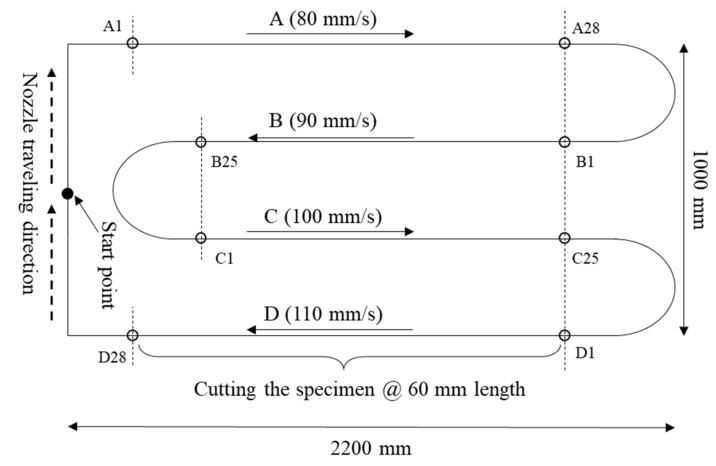
Printing design for experimental validation.

As shown in Figure 4, length (l) of 2200 mm and width (w) of the 1000 mm artifact with five layers was printed using nozzle traveling speeds of 80, 90, 100, and 110 mm/s. The height (h) of one layer during extrusion was set to 10 mm. Furthermore, all factors associated with layer formation, except the nozzle traveling speed, were controlled during fabrication.

#### 2.1.3. Measurement of the Printing Volume

The extrusion rate of the mortar was measured 10 min after mixing. A fixed rotation speed of 1.7 s^−1^ was used for the extruder, and the weight of the extruded mortar was measured three times at 60 s intervals. Additionally, the weight of the mortar extruded per minute (kg/min) was measured and converted into extrusion volume per second (mm^3^/s) based on the density of the material (2030 kg/m^3^) compared to the extrusion volume of the mortar.

### 2.2. Materials and Specimen

#### 2.2.1. Materials Used and Mixing

Three types of cementitious binders, a polycarboxylate-type high-water reduction agent (HWRA) produced by Dong-Nam corporation (Pyeongtaek-si, Gyeonggi-do, Republic of Korea), viscosity-modifying agent (VMA) produced by Dong-Nam corporation (Pyeongtaek-si, Gyeonggi-do, Republic of Korea), and sand, were used in this study. The three cementitious binders included the following: Type-1 ordinary Portland cement (OPC) produced by Asia Cement corporation (Yongin-si, Gyeonggi-do, Republic of Korea) with a specific gravity of 3.13 g/cm^3^ and initial and final sets of 263 and 360 min, respectively; Class-C-type fly ash (FA) produced by Maxcon cooperation (Boeun-gun, Chungchungbuk-do, Korea) with specific gravity and loss of ignition of 2.25 g/cm^3^ and 2.5%, respectively; and undensified silica fume (SF) produced by Elkem Korea (Seoul, Republic of Korea) with a SiO_2_ content of 91.3% and a residue rate of 45 µm for 4.4%. The specific gravity of the sand used was 2.59 g/cm^3^.

The mix proportions are listed in Table 1; 100 × 10^3^ cm^3^ (100 L) of mortar was mixed per batch. After placing the materials in a mixer, they were uniformly mixed without adding water for 1 min. The mixture was stirred three times each in clockwise and counter-clockwise directions for 1 min, followed by stirring for 6 min. The stirred mixture was immediately transferred into a pump for extrusion. The flow table test was performed following the ASTM standard C230 / C230M method [27] to evaluate the workability in the fresh state immediately after mixing, and a rheology test was performed as in previous studies [23]. The workability in the fresh state was 125 mm, and the yield stress was measured as 1464 Pa and plastic viscosity as 78.4 Pa∙s as rheological properties.

#### 2.2.2. Specimen Preparation

**Figure 5:**
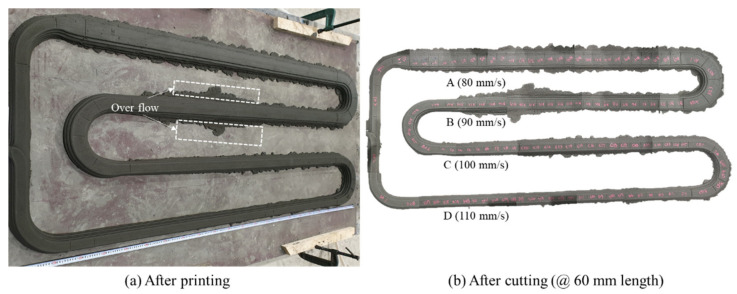
Printing artifact after (**a**) after printing; (**b**) after cutting (@ 60 mm length).

Figure 5a,b show the specimen, printed according to the artifact design shown in Figure 4, in a non-hardened state immediately after printing and cured after cutting to a 60 mm length, respectively. To prevent deformation of the specimen during cutting due to the fluidity of the material, the specimen was cut 1 h after printing and cured in a humidity chamber (25 °C ± 2 °C, relative humidity (RH) = 80%) after 24 h. To secure sufficient specimens, a minimum of 25 specimens were produced by cutting 60 mm layers.

Depending on the nozzle traveling speed, the specimens were named A (80 mm/s), B (90 mm/s), C (100 mm/s), and D (110 mm/s). The dimensions of the cut specimens were measured using Vernier calipers and used for interface analysis through X-ray CT analysis and to conduct compressive strength measurements.

#### 2.2.3. Compressive Strength Measurement

**Figure 6:**
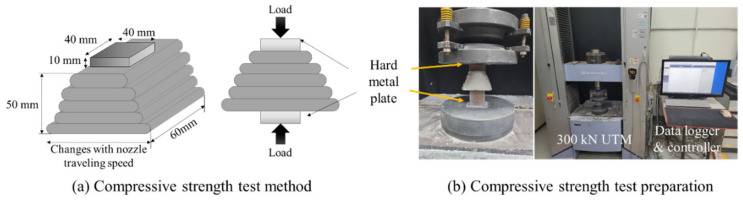
Compressive strength of the printed layer measured using a metal plate: (**a**) compressive strength test method; (**b**) compressive strength test preparation.

To evaluate the effect of the changes in the shape of the printed specimen on the material strength according to the nozzle traveling speed, the compressive strength was measured on the 3rd, 7th, 14th, and 28th day of curing. While printing the specimen, a 50 mm cube-shaped specimen was manufactured according to ASTM standard C109/C109M [28], and its compressive strength was measured at the same age as the printed specimen. A test method for compressive strength of laminated specimens was recently published in Korea. Unfortunately, this study was conducted before the publication of such a test method, so it could not be applied [29]. Given the inconsistent cross-sectional area of the printed specimen, a rigid, hard metal plate, with dimensions of 40 (w) × 40 (l) × 10 (h) mm, that does not undergo deformation was placed in the center of the specimen to prevent changes in strength, as shown in Figure 6a. As shown in Figure 6b, the compressive strength was measured using a 300 kN Universal Testing machine (Shimadzu, Kyoto, Japan) at a load rate of 0.5 kN/min.

### 2.3. Cross-section Measurement Method

#### 2.3.1. Apparent Size Measurement

The specimen length (60 mm) was measured using 20 specimens for four nozzle traveling speeds. Because it was difficult to visually distinguish the interface between layers in the cut cross-sections of the printed specimen, the width was measured at the part of each layer that protruded the most. Furthermore, the height of the layer was measured based on the valley between layers. Additionally, the apparent cross-sectional area of each layer was measured based on the width and height of each layer.

**Figure 7:**
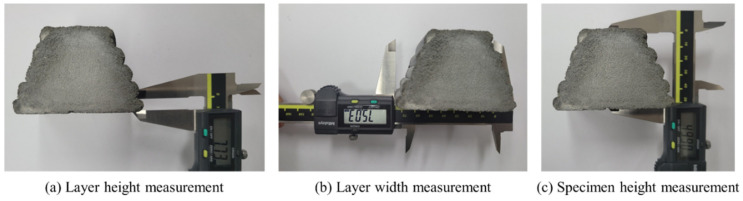
Size measurement using Vernier calipers: (**a**) layer height measurement; (**b**) layer width measurement; (**c**) specimen height measurement.

The apparent size was measured at 30 mm, which is the central point in the longitudinal direction, as shown in Figure 7. The height and width of the layer were measured at the center on the interface between layers and the outermost edge of each layer, as shown in Figure 7a,b, respectively. In addition, the height of the specimen was measured to determine whether the overall height of the specimen had changed, as shown in Figure 7c.

#### 2.3.2. Measurement of the Shape and Cross-Sectional Area of the Layer Using X-ray CT Equipment

An X-ray CT analysis was used on specimens printed at four different nozzle traveling speeds to measure the internal cross-sectional area. In order to secure the reliability of the cross-sectional analysis results between the appearance measurement results and the X-ray CT analysis, the specimen used for the apparent size measurement was used for the X-ray CT analysis. Five X-ray CT images were extracted from the printed specimens according to the nozzle traveling speed and used for cross-sectional analysis.

X-ray CT analysis and interpretation were performed using the SMX-225CT (Shimadzu Cooperation, Kyoto, Japan) and VG Studio MAX (Volume Graphics, Heidelberg, Germany), respectively. The environmental conditions inside the chamber were maintained at 25 °C and 35% RH. The cross-sectional area of the specimen was analyzed for each layer using five images, including two images in the 2.5 mm section before and after the center of the specimen, according to the direction of the nozzle movement.

**Figure 8:**
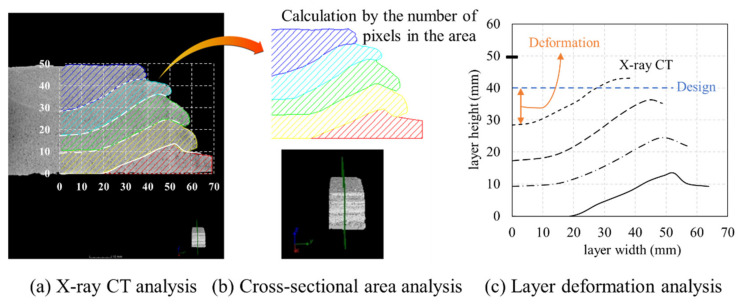
Relationship between extrusion volume and layer volume: (**a**) X-ray CT analysis; (**b**) cross-sectional area analysis; (**c**) layer deformation analysis.

Figure 8a shows the result of the X-ray CT analysis. After adjusting the scale of the X-ray CT image according to the height and width of the specimen, the cross-sectional area was analyzed by calculating the number of pixels along the length. As shown in Figure 8b, the number of pixels in the analyzed image was calculated, and the cross-section of the layer was calculated based on the boundary of the layer. In addition, the deformation of the layer was analyzed by evaluating the layer interface position of the specimen in the X-ray CT image, as shown in Figure 8c.

## 3. Results and Discussion

### 3.1. Printing Results

**Figure 9:**
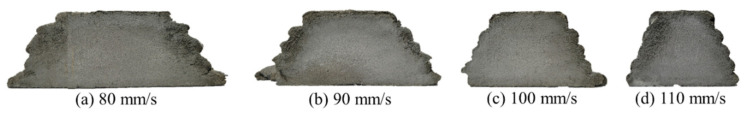
Printed specimen according to the nozzle traveling speed: (**a**) 80 mm/s; (**b**) 90 mm/s; (**c**) 100mm/s; (**d**) 110 mm/s.

The weight of the mortar extruded from the nozzle before printing was measured three times per minute, and the values were derived in terms of weight per minute (8.4 kg/min). The prints, as designed on the build platform in Figure 4, after measuring the extrusion weight, are shown in Figure 9. As the nozzle traveling speed increased from 80 to 110 mm/s, the layer width gradually decreased. Moreover, the material in the lowest layer of some sections did not maintain its original shape and spread sideways.

### 3.2. External Dimension Measurements

**Figure 10:**
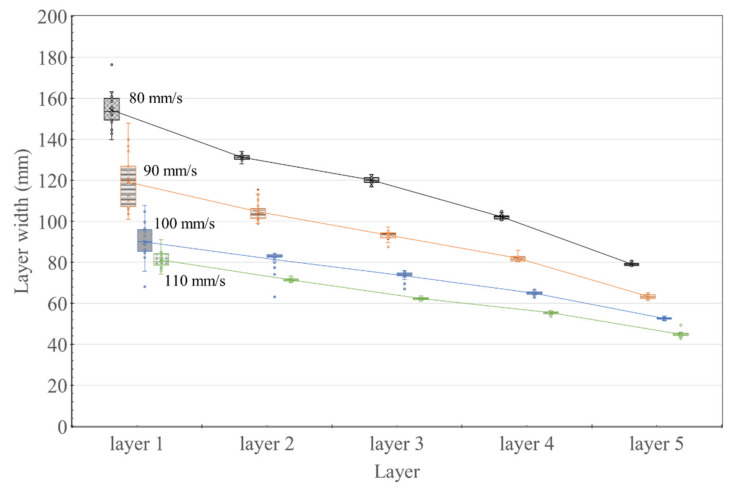
Variation in layer width measured using Vernier calipers for different nozzle traveling speeds.

**Figure 11:**
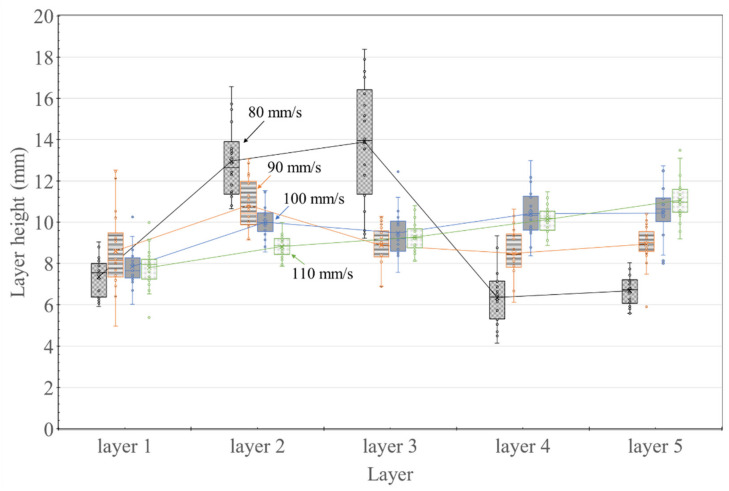
Variation in layer height measured using Vernier calipers for different nozzle traveling speeds.

Figure 10 and Figure 11 show the results of the external dimension (width and height of the layer) measurements of the 20 specimens at each nozzle traveling speed. As the nozzle traveling speed increased, the layer width decreased for each subsequent layer. Furthermore, the width of Layer 1 was approximately twice the width of Layer 5 for all nozzle traveling speeds, when considering that the increased pressure generated at the nozzle at the time of printing was transferred to the previously printed layer.

Figure 11 shows the variation in the layer height at different nozzle traveling speeds. When the nozzle traveling speed was 80–90 mm/s, no correlation was observed between the number of layers and the layer height. Conversely, at a traveling speed of 100–110 mm/s, the higher the layer location was, the higher the layer height was.

**Figure 12:**
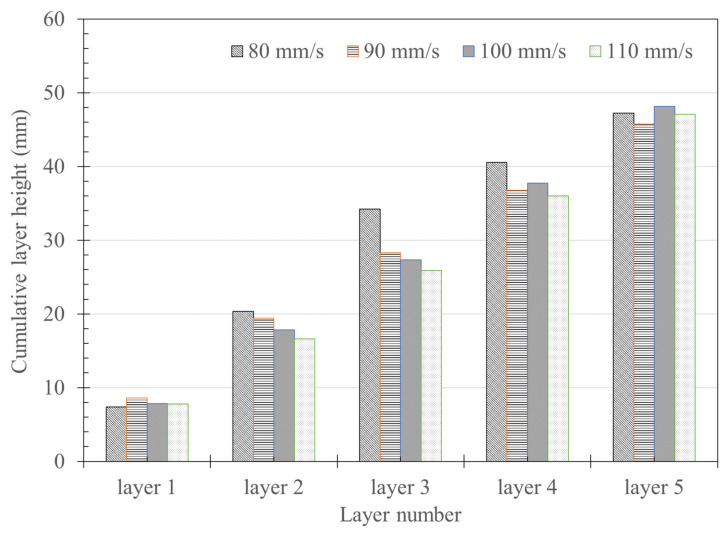
Relationship between extrusion volume and layer volume.

As shown in Figure 12, the results of accumulating the averaged measured heights in Figure 11 were similar to the height of the entire specimen. In addition, as seen from the results for Layer 5, the cumulative height was found to be similar in general, regardless of the nozzle traveling speed.

As shown in Figure 10, as the nozzle traveling speed increased, the layer width decreased, and the layer width decreased according to the layer printing order (from Layer 1 to Layer 5). When the nozzle traveling speed is increased, the contact time to form a layer at the printing cross-section is shortened. Therefore, it is predicted that an increase in the nozzle traveling speed while the extrusion amount is constant reduces the layer’s width. In addition, it is judged that the lower the layer, the greater the deformation because it receives loads and pressures from the upper layers.

The layer height measurement results in Figure 11 showed a different trend from the layer width measurement results. When the nozzle traveling speed was 80 mm/s, it was larger than the layer design height of 10 mm in the second and third layers. On the other hand, it appeared as 7 mm in Layers 4 and 5. However, when the nozzle moving speed was 110 mm/s, the layer height gradually increased according to the layer printing order. The height of Layer 1 was fabricated almost the same, regardless of the nozzle traveling speed. However, as the nozzle traveling speed increased, the Layer 2 height gradually decreased. The cause of this trend is predicted to be due to the interaction between the build platform and the material extrusion pressure generated from the nozzle. Since the first layer is in direct contact with the build platform, where no deformation occurs, the deformation applied to the material was the same regardless of the nozzle traveling speed. However, in the case of Layer 2, as the nozzle traveling speed increased, the layer height was fabricated lower. This is thought to be because, as the moving speed of the nozzle is smaller, the material pressure is transmitted to Layer 1 and the build platform and then rebounds and rises again. However, as shown in Figure 9, the interface of the layers cannot be checked visually, so this should be confirmed through X-ray CT analysis.

As shown in Figure 12, although the heights of each layer were different, the result of adding up to Layer 5 by accumulating the average value of each layer height was approximately 50 mm, the design height of the specimen. It was found that fabricating layers is possible according to the set nozzle height. However, it is expected that the height gradually increases and the width gradually increases according to the printing order to compensate for the phenomenon that the height of the lower layer decreases and the width becomes wider during the printing process. However, based on the previous Figure 11 and Figure 12, it is predicted that the deformation of the material due to the pressure transferred from the nozzle to the layer during extrusion of the material was greater than the load of the material of the upper layer.

**Figure 13:**
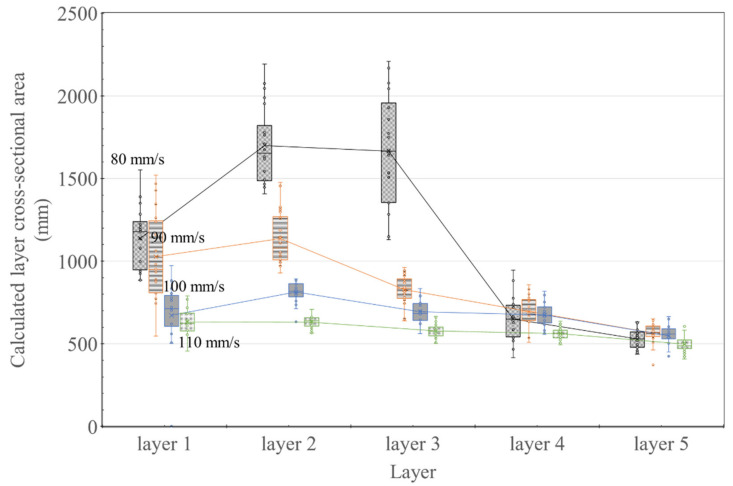
Variation in the layer cross-sectional area size (measured using vernier calipers) for different nozzle traveling speeds.

Figure 13 shows the layer cross-sectional area results calculated by multiplying the width and height of the layer, assuming a rectangular cross-section. The area calculation results for up to the third layer for nozzle traveling speeds of 80 and 90 mm/s showed abnormally high values compared to the rest of the results shown in Figure 13. However, from the fourth layer onward, the values were similar to those for 100 and 110 mm/s. While extrusion at 100 and 110 mm/s resulted in an increase in width and decrease in the height of the bottom layer, the cross-sectional area remained the same regardless of the layer.

**Figure 14:**
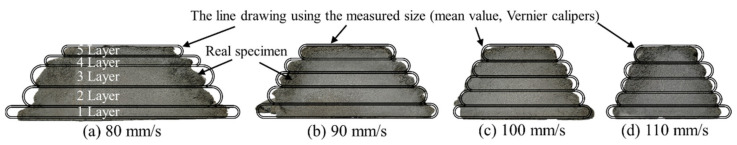
Printed specimen and visualized shape of the layers measured using a Vernier caliper: (**a**) 80 mm/s; (**b**) 90 mm/s; (**c**) 100 mm/s; (**d**) 110 mm/s.

Figure 14 shows a photograph of the printed specimen and the mean value of the width and height of the layer measured using Vernier calipers (line drawing). While the external appearance could be confirmed for the actually printed specimen, it was difficult to visually locate the interface. Therefore, to compare and examine the shape and cross-sectional area of the layer interface with those acquired in the X-ray CT analysis, the shape of the layer interface was analyzed using the average measurement result.

As shown in Figure 14, the figure drawn using the average value was similar to the size of the printed specimen. Considering the results for 80 mm/s in Figure 14a, the second and third layers had a relatively wide cross-sectional area compared to that of the other layers. In addition, while the cross-sectional area of Layer 1 increased, it decreased in Layers 4 and 5. However, according to previous studies, the extrusion amount decreases with time by approximately 5% over 1800 s [24]. Based on our experimental results, the effect of extrusion reduction on the layer cross-sectional area is insignificant when printing five layers.

If the amount of extrusion does not affect the layer cross-sectional area, it should maintain the same area at the same nozzle travel speed. However, as shown in Figure 14, the layer cross-sectional area generally decreases, indicating that the cross-sectional area calculated using Vernier calipers cannot represent the total cross-sectional area, and the shape of the actual layer is not parallel to the build platform.

### 3.3. Analysis of the Layer Width and Height Using X-ray CT Analysis

**Figure 15:**
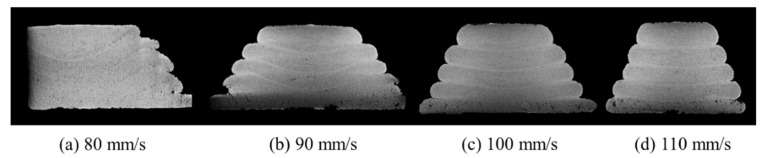
X-ray CT images of the specimens and their sizes for different nozzle traveling speeds: (**a**) 80 mm/s; (**b**) 90 mm/s; (**c**) 100 mm/s; (**d**) 110 mm/s.

After measuring the layer, the dimensions of the specimen printed according to the experimental variables and its internal cross-section were evaluated using the X-ray CT analysis method, as shown in Figure 15. The results showed that the internal layer was not in level with the built platform, owing to the deformation in the middle of the layer. This deformation was more noticeable at 80 mm/s, where the nozzle speed was relatively slow. In particular, the height of the area in the topmost layer, where deformation occurred the most at 80 mm/s, was the same as that in the interface of the second layer from the top at 110 mm/s. This sagging gradually decreased as the nozzle traveling speed increased.

**Figure 16:**
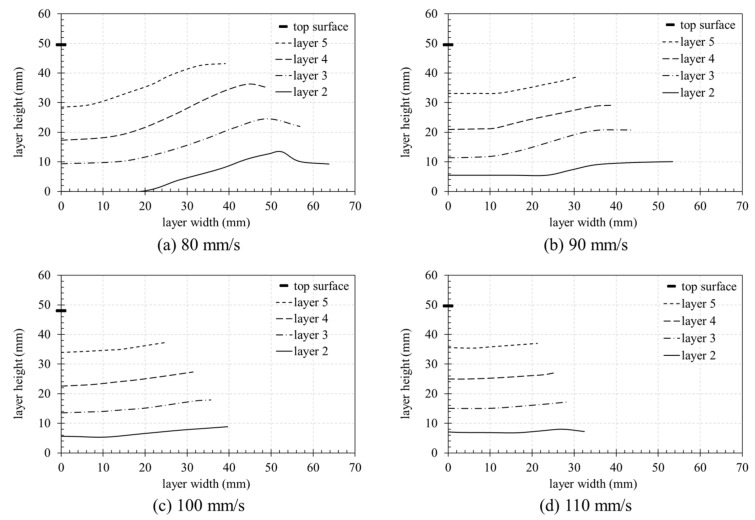
Interface analysis of the specimens using X-ray CT analysis: (**a**) 80 mm/s; (**b**) 90 mm/s; (**c**) 100 mm/s; (**d**) 110 mm/s.

Figure 16 shows the height of the layer interface and the distance from the center to the edge of the cross-section. As shown in Figure 16a–d, the height of the top surface was the same irrespective of the nozzle traveling speed. Furthermore, there was little deformation, owing to an error of less than 2 mm (compared to the 50 mm design height) of the nozzle when printing five 10 mm layers. At 80 mm/s, the height of the second layer was approximately zero in the center because the extrusion pressure, generated from the nozzle when the upper layer is laminated in the center part, was not delivered to the lower part but to both sides, owing to the rigidity of the built platform in the first layer. Moreover, the height of the interface of the second layer was greater than the 10 mm design height, owing to the increase in material pushed sideways from the center.

The height of the interface at the center of the specimen was the lowest and increased gradually from the center to the edge. At 80 mm/s, the height of the interface was significantly higher than the design height at the edge. However, the height of the interface between Layers 1 and 2 was similar for the nozzle traveling speeds of 90, 100, and 110 mm/s, with both layers having a threshold level that was no longer deformed at a certain section in the center, as shown in Figure 16. In addition, as shown in Figure 16b–d, the visibility of the interface between the first and second layers decreased compared to the other interfaces, owing to the disturbance in the layer boundary.

**Figure 17:**
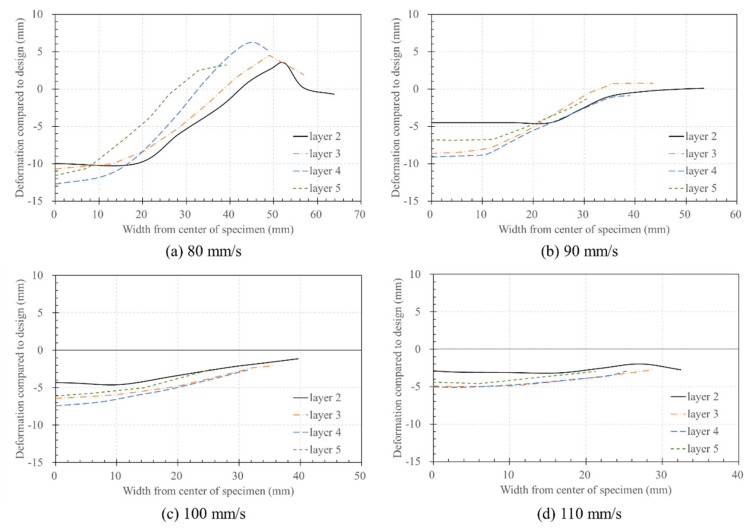
Deformation of the layers according to nozzle traveling speed: (**a**) 80 mm/s; (**b**) 90 mm/s; (**c**) 100 mm/s; (**d**) 110 mm/s.

**Figure 18:**
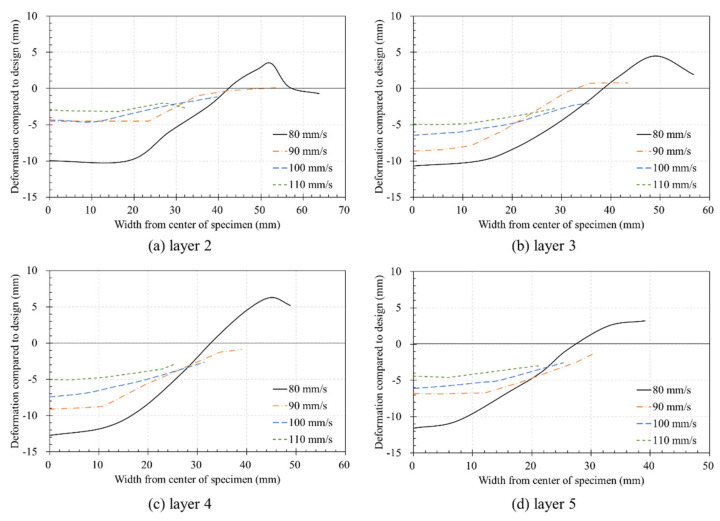
Deformation of the layers according to printing order: (**a**) layer 2; (**b**) layer 3; (**c**) layer 4; (**d**) layer 5.

Figure 17 and Figure 18 show the difference between each layer and the designed layer height, respectively. Based on the originally designed height (e.g., 10 mm for two layers and 20 mm for three layers), the graph shows negative and positive values when the height decreased and increased, respectively. Figure 17 shows the deformed height of the layer according to the nozzle traveling speed. As the nozzle traveling speed increased, the deformation of the layer decreased. In all cases, the central part was lower than the designed height, which gradually increased as the distance from the central part increased. In particular, at 80 mm/s, the layer height increased as the measurement width increased from the center to the edge, which became higher than the designed height in the 28–42 mm section, as shown in Figure 17a. This can be attributed to the fact that the material in the squeezed bottom layer rises to the side, when considering it is pressed down, as shown in Figure 16. This could have been mistaken for an increased height.

Figure 18 shows the deformation of the layers according to their printing order. In Figure 18a, all values except those for a nozzle traveling speed of 80 mm/s were higher than −5 mm. However, as shown in Figure 18b,c, the deformation increased as the number of layers increased. As shown in Figure 16, while the built platform at the bottom could no longer be lowered, when considering that deformation did not occur, the deformation was significant in Layers 3 and 4 while pressing the lower layer. The combined height of the five layers was slightly higher than that of the four layers, which can be attributed to the fact that Layer 5, located at the top, did not print a sixth layer, due to which no additional deformation occurred.

Through the results of measuring the degree of deformation of the layer interface through Figure 16 and Figure 17, the results of the appearance investigation using Vernier calipers can be explained. For example, in Figure 11, in the case of a nozzle moving speed of 80 mm/s, the height of Layers 2 and 3 was significantly higher. In Figure 16a, printed layer height did not reach the design height of each layer (design height: 10 mm at layer 2; 20 mm at layer 3; 30 mm at layer 4), but the height gradually increased toward the edge from the centerline (layer width 0 mm on the x-axis) of the specimen. In the case of Layers 2 and 3, 40 mm from the centerline was higher than the layer design height. It is expected that the intense pressure generated by the nozzle is transmitted to the edge of the layer, and the deformation of the material affects not only the vertical direction but also the left and right directions. As the layer is transferred in the left and right direction due to the pressure transferred to the edge, it is predicted that the accumulated pressure rises upward at the edge of the layer.

Furthermore, the reason that the height of Layers 2 and 3 is the highest in Figure 11 and the height of Layer 4 is lower can be explained through Figure 16a. Since the appearance investigation used the method of measuring the height of the layer at the edge of the specimen, deformation in the center of the layer cannot be reflected. Therefore, it appears as a result of measuring between the edge points in each layer height graph, as shown in Figure 16a. Thus, the height of Layer 4 in Figure 11 is the same as the edge height difference between Layers 4–5 in Figure 16a. As a result, it is judged that the appearance inspection method is not a suitable method for confirming the cross-sectional information when the layer is deformed.

As shown in Figure 17, the most considerable deformation occurs in the center of the layer, and the deformation gradually recovers toward the edge. In particular, in Figure 17a, the amount of deformation increases toward the edge, and the deformation occurs in the upward direction after a certain section. These results can support the claim that the pressure of the nozzle is transmitted from side to side, and the material rises to the top.

Furthermore, the size of the deformation amount on the centerline appeared in the order of Layer 4 → 3 → 5 → 2. The cause of the appearance of the order of the deformation amount of the layer can be expected due to the influence of the build platform. The build platform, which is a rigid, deformation-free build platform, is expected to transfer the extrusion pressure of the material back to the material without absorbing it. In particular, in the case of Layer 2, it is predicted that the smallest deformation appeared because the pressure generated from the nozzle was offset or supported by the build platform.

The influence of the build platform is expected to have affected up to Layer 4. When the Layer 5 result is excluded, the deformation occurs in the order of Layer 4 → 3 → 2, which is judged because the greater the distance from the build platform, the smaller the effect of the build platform’s supporting resistance. However, in the case of Layer 5, there was no pressure transmitted from the upper layer, and compensation for the height difference between the printing height of Layer 4 and the design height of the nozzle of 50 mm was required, so the amount of deformation is expected to be small.

### 3.4. Layer Cross-Sectional Area Calculation Using X-ray CT Analysis

**Figure 19:**
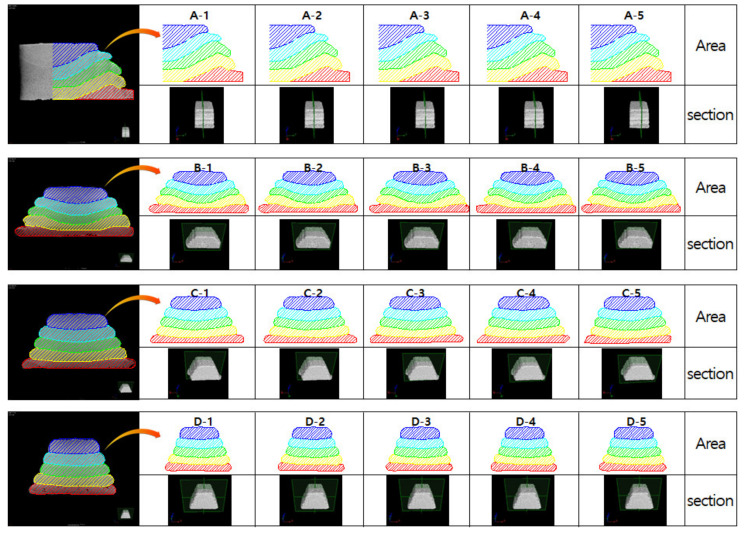
Analysis method for the cross-sectional area of the specimens using X-ray CT analysis.

To calculate the cross-sectional area using the cross-sectional shape extracted from the X-ray CT analysis, the areas were expressed according to the surface of each layer, as shown in Figure 19. However, at a nozzle traveling speed of 80 mm/s, the values obtained via X-ray CT analysis exceeded the maximum possible size of the specimen. Therefore, the CT analysis of the 80 mm/s specimen was summarized by analyzing the left and right sides based on the centerline.

**Figure 20:**
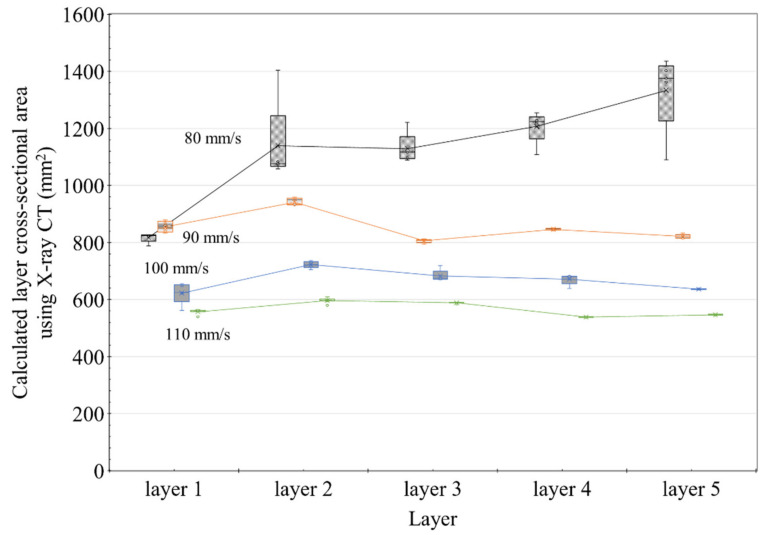
Analysis results for the cross-sectional areas of the specimens using X-ray CT analysis.

Figure 19 is the actual result of expressing the area of each layer in different colors after extracting the cross-section with X-ray CT, and the graph of Figure 20 was derived based on this result. In Figure 19, Area represents the result of expressing the cross-sectional area of each layer in color, and section describes the location of the cross-section from which the image was extracted from the specimen. The calculated area of the layer was expressed as a box–whisker plot, as shown in Figure 20. The results showed that all except for the 80 mm/s series had smaller boxes, indicating that the distribution range of the calculated areas was relatively small.

Figure 20 shows the cross-sectional area of the layer calculated from the X-ray CT results. The results showed that the cross-sectional area was almost constant based on the nozzle traveling speed, irrespective of the printing order of the layers. As shown in Figure 20, unlike the results measured with Vernier calipers shown in Figure 13, the results showed a fairly constant level. However, the cross-sectional area of the 80 mm/s specimen gradually increased according to the printing order of the layers. Nonetheless, the results shown in Figure 13 are quite similar to those shown earlier.

Figure 19 is the actual result of expressing the area of each layer in different colors after extracting the cross-section with X-ray CT, and the graph of Figure 20 was derived based on this result. In Figure 19, Area represents the result of expressing the cross-sectional area of each layer in color and section as shown in Figure 20. In the analysis result using X-ray CT, it can be seen that the cross-sectional area of the layer decreases as the nozzle traveling speed increases. In addition, in the case of nozzle traveling speeds of 90, 100, and 110 mm/, it can be seen that the deviation of the cross-sectional area measurement result was remarkably reduced compared with Figure 13. Even at a nozzle traveling speed of 80 mm/s, the deviation was reduced compared to Figure 13, except for the Layer 1 result, and stable results were obtained. Through Figure 20, it can be proved that the cross-sectional area of the layers is the same regardless of the printing order of the layers when printing using the same amount of extrusion. However, it was confirmed through the nozzle traveling speed of 80 mm/s in Figure 19 that the lower layer could be destroyed beyond the level of deformation due to the extrusion pressure of the material if the nozzle traveling speed was slower than a certain threshold. The destroyed layer is pushed toward the edge of the layer, resulting in the loss of the material forming the layer. In Figure 20, the small cross-sectional area of Layer 1 is measured because the layer material is lost to the outside. As a result of Figure 20, it is thought that Lee et al. [23] can explain the layer overflow phenomenon that occurred in the previous study.

### 3.5. Comparison of the Layer Cross-Sectional Area Following the Measurement Method

**Figure 21:**
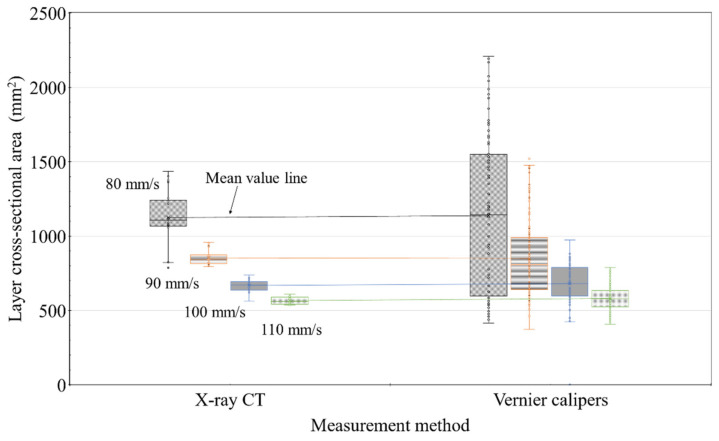
Analysis of the cross-sectional area measured using X-ray CT analysis and Vernier calipers.

Figure 21 shows a comparison of the cross-sectional areas analyzed using X-ray CT analysis and the apparent cross-sectional areas measured using Vernier calipers by aligning the measurements of all layers with the nozzle traveling speed. Although a difference was observed in the number of samples used in the analysis, the results obtained via X-ray CT analysis were expected to be more precise.

Table 2 summarizes the median, mean, and standard deviation of the results shown in Figure 21. The median and mean values in the two sets of results were found to be almost the same, indicating that the layer measurements were highly reliable. However, the standard deviation obtained using the apparent measurement was 2.7–5.3 times higher than that obtained from the X-ray CT analysis, indicating a difference in measurement precision.

Figure 21 and Table 2 show the results of calculating the cross-sectional area of the layer using both the external examination method and the X-ray CT method. When comparing the cross-sectional area measurement results of Layers 1–5 only at the nozzle traveling speed regardless of the layer printing order, the deviation of the two results is different, but the average value is almost similar. As a result, it was experimentally confirmed that the nozzle traveling speed is the main factor determining the cross-sectional area of the layer.

As a measurement method, it was confirmed that it was effective to analyze the shape of the cross-section of the layer and the position of the interface using the X-ray CT technique and showed relatively minor errors. On the other hand, in the case of the exterior investigation method, the position of the cross-section and the interface of the layer could not be grasped. The reliability of the measurement results was low, and the deviation was significant. Furthermore, although the reliability of the cross-sectional area calculation results according to the printing order was low, it was concluded that the average value calculated using 100 data according to the nozzle traveling speed was reliable nonetheless.

### 3.6. Compressive Strength Measurement Results

**Figure 22:**
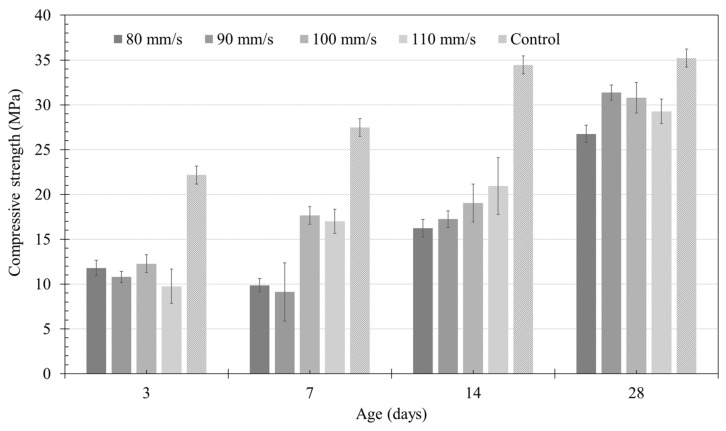
Compressive strength by material age according to different nozzle traveling speeds.

The compressive strengths of the five specimens were measured for each series according to the material age. Figure 22 shows the average calculated values. For the control specimens, the same material was cured under the same curing conditions, and the compressive strengths were found to be 22.2, 27.5, 34.5, and 35.2 MPa according to the material age. Compared to the extrusion specimens, this showed a higher compressive strength by minimum and maximum strength values of 3.8 and 8.5 MPa, respectively, on a 28-day basis. Under the same conditions, the compressive strength was relatively low in the printed specimens, which was also observed in a previous study [18]. Although there could be various reasons for imbalance in the shape and existence of interlayer interfaces, the cause has not yet been identified. However, the biggest difference in the quality of the printed specimen is expected to be in terms of the homogeneity of the material when compared to mold–cast specimens.

Differences were observed in the compressive strengths of printed specimens according to the material age. However, upon comparing the compressive strength of specimens A and B having a material age of 28 days, their average compressive strength differed by 4.6 MPa. The compressive strength according to the nozzle traveling speed was not determined in this study.

## 4. Conclusions

In this study, we proposed a method to analyze the cross-sectional area of layers in a specimen developed using 3D printing technology. Experimental validation was performed through X-ray CT analysis. The experiments were conducted only for the application of the lamination method using a circular nozzle, and meaningful experimental results were derived. The results derived from this study can be summarized as follows,

- The X-ray CT method could obtain more accurate results than the appearance investigation method to analyze the cross-sectional area of the deformed layer and the shape of the interface. Through the X-ray CT analysis, it was possible to analyze the shape of the interface due to the deformation of the layer, which could not be confirmed in the appearance investigation. Although the deformation of the layer occurred, the cross-sectional area was maintained regardless of the printing order. The appearance investigation method could not analyze the layer interface. Still, the cross-sectional area of the layer was almost similar to the X-ray CT analysis result when the average value of 100 data was used.
- Four nozzle traveling speeds (80, 90, 100, and 110 mm/s) were set for the experimental validation, and printing was performed. Upon measuring the apparent size using Vernier calipers, a significant difference was observed in the area depending on the printing order of the layer. Particularly, a significant variation was observed in Layers 1–3 of the test specimen, showing relatively slow nozzle traveling speeds of 80–90 mm/s. In contrast, the analysis of the cross-sectional area showed a similar layer area regardless of the printing order of the layers. However, the cross-sectional area of Layer 1 of the 80 mm/s specimen was 60%–70% compared to the other layers, which can be attributed to material loss due to overflow.
- The nozzle traveling speed affects the cross-sectional area of the layer when the extrusion amount of the material is the same. If the nozzle traveling speed is slow, the time required to pass through any one point of the layer becomes more extended. The extrusion amount increases because the extrusion time of the material becomes longer. Therefore, since the nozzle traveling speed becomes a significant factor in determining the layer’s cross-sectional area, if the nozzle’s traveling speed is slowed, the cross-sectional area of the layer increases.
- Moreover, it was found that the nozzle traveling speed affected the deformation of the layer. The same amount of material extruded means the same pump pressure applied to the material, and it can be assumed that the material extrusion pressure at the nozzle is the same. The slower the nozzle traveling speed, the longer the amount of time affected by the extrusion pressure of the material at any point in the layer. As a result of X-ray CT, the layer interface showed a tendency to become lower at the center of the specimen and higher towards the edge. As such, it is assumed that the shape of the layer interface was formed radially from the center because the extrusion pressure of the material was generated from the nozzle and transmitted radially to the layer. In particular, when the nozzle traveling speed is lowered below a specific critical value, the lower layer is destroyed due to the extrusion pressure, and the material is pushed to the edge.
- Although the layer cross-sectional area varied according to different nozzle traveling speeds, no significant changes were observed in the strength according to the nozzle traveling speed. However, the strength of the printed specimen was lower than that of the mold–cast specimen, as shown in previous studies. Therefore, it is necessary to study the correlation between the surface and compressive strength of printed specimens in future work.

Although the layer cross-sectional area was analyzed using measurement methods, the extrusion pressure in the nozzle causes deformation in the surface of the layer, and the phenomenon that causes the spreading of the layer is not identified. Therefore, it is necessary to study the phenomenon of surface deformation caused by the extrusion pressure in the nozzle in future studies. Furthermore, considering that this study only dealt with straight-lined printed shapes, further research needs to be conducted on specimen shapes generated by changing the nozzle direction.

## Figures and Tables

**Table 1:** Mix proportions (kg/m^3^).

W/B	Water	OPC	FA	SF	Sand	HWRA	VMA
0.28	214.59	533.28	153.41	76.70	1054.69	15.27	0.76

**Table 2:** Comparison of the measurement results obtained using Vernier calipers and X-ray CT analysis.

(mm^2^)	X-ray CT Analysis			Vernier Calipers		
Series	Median	Mean	Standard Deviation	Median	Mean	Standard Deviation
80 mm/s	1108.6	1125.5	195.2	1142.4	1136.7	537.8
90 mm/s	842.1	854.1	49.2	808.4	850.3	260.9
100 mm/s	671.7	667.3	41.0	681.4	680.6	137.0
110 mm/s	561.3	565.7	24.3	579.2	580.5	77.5

## Data Availability

The data used to support the findings of this study are included within the article.
